# Chemical, Physical and Biological Approaches to Prevent Ochratoxin Induced Toxicoses in Humans and Animals

**DOI:** 10.3390/toxins2071718

**Published:** 2010-07-01

**Authors:** János Varga, Sándor Kocsubé, Zsanett Péteri, Csaba Vágvölgyi, Beáta Tóth

**Affiliations:** 1 Department of Microbiology, Faculty of Science and Informatics, University of Szeged, Közép fasor 52, H-6726 Szeged, Hungary; Email: shigsanyi@gmail.com (S.K.); zspeteri@gmail.com (Z.P.); csaba@bio.u-szeged.hu (C.V.); 2 PannonPharma Company, Mária dűlő 36, H-7634 Pécs, Hungary; 3 Cereal Research Non-Profit Limited Company, Alsókikötő sor 9, H-6726 Szeged, Hungary; Email: beata.toth@gabonakutato.hu (B.T.)

**Keywords:** ochratoxin, detoxification, adsorption, spoilage

## Abstract

Ochratoxins are polyketide derived fungal secondary metabolites with nephrotoxic, immunosuppressive, teratogenic, and carcinogenic properties. Ochratoxin-producing fungi may contaminate agricultural products in the field (preharvest spoilage), during storage (postharvest spoilage), or during processing. Ochratoxin contamination of foods and feeds poses a serious health hazard to animals and humans. Several strategies have been investigated for lowering the ochratoxin content in agricultural products. These strategies can be classified into three main categories: prevention of ochratoxin contamination, decontamination or detoxification of foods contaminated with ochratoxins, and inhibition of the absorption of consumed ochratoxins in the gastrointestinal tract. This paper gives an overview of the strategies that are promising with regard to lowering the ochratoxin burden of animals and humans.

## 1. Introduction

Ochratoxin A (OTA) is a mycotoxin that contaminates different plant products, including cereals, coffee beans, nuts, cocoa, pulses, beer, wine, spices, and dried vine fruits [1]. Ochratoxins are cyclic pentaketides: dihydroisocoumarin derivatives linked to an L-phenylalanine moiety. OTA was first discovered in 1965 in an *Aspergillus ochraceus* isolate [2]. Since then, several *Aspergillus* and *Penicillium* species have been described as producers of this mycotoxin [3]. OTA proved to exhibit nephrotoxic, immunosuppressive, teratogenic, and carcinogenic properties [4]. Several nephropathies affecting animals as well as humans have been attributed to OTA; e.g., this mycotoxin is the etiological agent of Danish porcine nephropathy and renal disorders observed in other animals [5]. In humans, OTA is frequently cited as the possible causative agent of Balkan endemic nephropathy, a syndrome characterized by contracted kidneys with tubular degeneration, interstitial fibrosis, and hyalinization of the glomeruli [6,7]. In 1993, the International Agency for Research on Cancer (IARC) classified OTA as a possible human carcinogen (Group 2B) and concluded that there was sufficient evidence in experimental animals, but inadequate evidence in humans for the carcinogenicity of OTA [8]. OTA has also been suggested to play a role in chronic karyomegalic interstitial nephropathy and chronic interstitial nephropathy in Tunisia [9], urothelial tumors (end-stage renal disease) in Egypt [10], and testicular cancer [11]. 

Maximum levels for OTA in commodities have been set by Commission Regulation (EC) for several agricultural products destined to be used as food or feed ingredients [3]. There are also national laws and regulations in the European Union. Several strategies have been proposed to prevent the toxic effects of mycotoxins in general, and of ochratoxins in particular, in foods and feeds [12]:

(i) prevention of mycotoxin contamination;
(ii) decontamination or detoxification of foods contaminated with mycotoxins;
(iii) inhibition of the absorption of consumed mycotoxin in the gastrointestinal tract.

## 2. Prevention of Mycotoxin Contamination

### 2.1. Preharvest management

Prevention of growth and mycotoxin production in fungi from the field is usually considered the best approach to impede the harmful effects of mycotoxins on animal and human health. As mycotoxin producing molds can usually only colonize damaged parts of plants, crops must be protected against damage caused by either mechanical processes or insects. Besides, inoculum sources, such as weeds or agricultural residues, should be minimized to avoid contamination. Regarding the plants themselves, stress should be reduced, and good agricultural practices (GAP) should be followed including crop rotation, and culture and harvest in the appropriate seasons and conditions [13]. Indeed, Rousseau and Blateyron [14] also emphasized that the occurrence of OTA in wine may be decreased via appropriate vineyard management by about 80%.

For lowering pre-harvest contamination, treatment of field crops with fungicides is the traditional technique. The effect of fungicides on mold growth and mycotoxin biosynthesis is affected by several factors, including their chemical nature, rate of application, crop type, fungal species, and storage conditions [12]. The organophosphate fungicide, dichlorvos, was found to inhibit OTA production of *A. sulphureus*, *P. verrucosum*, and *A. ochraceus* [15,16]. Another fungicide, iprodione, has successfully been used in agricultural commodities to prevent the growth of various fungal species, including OTA producers [12], and was found to be able to decrease OTA production of *A. westerdijkiae* [17]. The effects of fungicide treatments on the OTA content of wines have been examined in several laboratories. In earlier studies, combinations of Euparen (a sulfamide type fungicide) and Mycodifol [18], or captan, [19] were found to be effective against black aspergilli, which colonize grape berries. Recently, Lo Curto *et al.* [20] observed that the application of some pesticides, such as Azoxystrobin (a strobilurin derivative) or Dinocap (a dinitrophenyl derivative), in combination with sulfur, effectively decreased the OTA content of wines. Carbendazim and Chorus were found to be ineffective in controlling sour rot caused by aspergilli [21]. However, the application of another pesticide, Switch, led to a significant decrease in incidence of black aspergilli on grapes, as confirmed in field trials carried out in France, Spain, Greece, and Italy [21,22,23]. The fungicide Switch contains cyprodinil and fludioxonil, which belong to the pyrimidine and pyrrolnitrin classes of fungicides, respectively [21]. The observation that fludioxonil can be used against aspergilli is not surprising, since pyrrolnitrin was found previously to be effective against black aspergilli [24]. In another study, the fungicides Switch, Scala (containing the pyrimidine fungicide pyrimethanil), and Mikal (containing fosetyl-Al and the dicarboximide folpel) were found to be the most effective for lowering fungal colonization and the OTA content of wines [25]. Several other fungicides have been shown to be active in reducing either fungal growth or OTA levels in grapevine, including mepanipyrim, pyrimethanil, fluazinam, and iprodione [26]. Belli *et al.* [23] examined the effect of 26 fungicides on *A. carbonarius* infection and OTA production in synthetic medium and on grapes, and found that both infection and OTA production were reduced when using cyprodinil plus fludioxonil, azoxystrobin, and penconazole. However, it should be mentioned that some fungicides were found to stimulate OTA production in grapes [27]. For example, carbendazim has been found to reduce fungal biota, but to stimulate OTA production [28], while fenhexamid, mancozeb, and copper hydroxide plus copper also enhanced infection and OTA production in grapes [23]. Recently, fusapyrone, an antifungal compound produced by *Fusarium semitectum*, and natamycin were found to be effective in controlling OTA-producing aspergilli and OTA levels in vineyards [29,30].

The application of insecticides that work against vectors of ochratoxin producing fungi can also be used successfully to lower OTA levels in grapes and coffee. In grapevine, a good correlation between damage caused by the grape-berry moth (*Lobesia botrana)* and OTA content has been found in grape berries, due to the contribution of *L. botrana* to berry wounds and fungal spore dissemination [31]. Field trials confirmed that a successful control of *L. botrana* using either biological methods or insecticides reduced fungal infection and OTA accumulation in grapes [22,26]. Moreover, insecticide treatment against *L. Botrana*, in combination with fungicides, contributed significantly to a reduction of OTA levels in the field [22]. Research carried out at the Interprofessionnel de la Vigne et du Vin France (ITV France) indicated that larvae of grape moth *Cochylis* sp. also act as vectors for the conidial dispersal of OTA-producing fungi [32]. A strict correlation was observed between the number of perforations caused by these larvae and OTA concentrations in grapes. Consequently, researchers at the Institut Coopératif du Vin (ICV) successfully used the insecticides Lufox (carbamate type insecticide containing luferunon and fenoxycarb), Decis (a pyrethroid insecticide containing delthametrin), and Bt (*Bacillus thuringiensis*) for lowering the OTA content of wines [33]. *Bacillus thuringiensis* was also found to significantly inhibit the growth of OTA-producing fungi on grapes in another study [34].

In coffee, the coffee berry borer (also called broca), *Hypotenemus hampei*, has been shown to be a vector of *A. ochraceus* [35]. Insecticides controlling these insects could be successful in lowering the OTA contamination of coffee beans. However, such trials have not been carried out, partly due to the resistance of the coffee berry borer against some pesticides (e.g., Endosulfan), and also to the high toxicity of these insecticides. Other approaches are also used to control the coffee berry borer in the field, including cultural and manual methods, traps, or biological control using toxin-producing *Bacillus thuringiensis* strains [36], entomopathogenic fungi (e.g., *Beauveria bassiana*), or the insect parasitoids *Cephalonomia stephanoderis* or *Prorops nasuta* [37]. Such approaches could also reduce the OTA content of coffee beans. However, the insect parasitoid *Prorops nasuta*, which has been introduced from Africa to many coffee-producing countries in an attempt to control the coffee berry borer, has also been shown to carry another OTA producing mold, *Aspergillus westerdijkiae* [38]. These results raise the possibility that this insect parasitoid might be disseminating an ochratoxin-producing fungus in coffee plantations. Promising results were also obtained in grape fields using yeasts as a biological control agents. In particular, good results were obtained with *Cryptococcus laurentii* and *Aureobasidium pullulans*, and with a strain of *Hanseniaspora uvarum* [26]. In field trials, an *Aureobasidium pullulans* strain was found to be an effective biocontrol agent of *A. carbonarius*, reducing both severity of *Aspergillus* rots and OTA accumulation in wine grapes [39]. This isolate was also able to degrade OTA in *in vitro* experiments. Other epiphytic yeasts, including *Candida guilliermondii*, *Acremonium cephalosporium* [40], *Issatchenkia orientalis*, *Metschnikowia pulcherrima*, *Issatchenkia terricola,* and *Candida incommunis* [41] have also been found to be effective in preventing rots caused by *Aspergillus niger* in wine grapes. Antifungal metabolites isolated from the culture fluids of *Bacillus pumilus* inhibited the production of OTA [42]. 

Biological control using non-toxigenic *Aspergillus niger* isolates against toxin-producing black aspergilli has also been applied successfully in grapes [43]. On the other hand, coinfection with toxigenic black aspergilli and either *Penicillium janthinellum* or *Eurotium amstelodami* increased OTA content. A biocontrol rhizobacterial strain of *Bacillus subtilis* AF 1 has also been found to lyse *A. niger* hyphae, and was suggested to be used as a biological control agent against black mold [44].

Plant breeding is traditionally used to improve the resistance of the host plants to fungal infection. Such attempts are promising, e.g., in the case of *Fusarium* infection of wheat and corn. Kernels of several varieties of wheat, rye, and barley were found to have different resistance levels to fungal attack and OTA accumulation. Thus, varieties with stronger resistance to fungal invasion during storage could be selected [45]. However, to our knowledge, breeding has not been used to increase the resistance of cereals to OTA accumulation. There is also limited information on the susceptibility levels of grape varieties to infection and on OTA accumulation caused by black aspergilli [46]. In *in vitro* experiments, 3 of the 12 tested varieties, namely ‘‘Bianco di Alessano’’, ‘‘Pampanuto’’, and ‘‘Uva di Troia’’, showed low OTA contamination after artificial infection with a mixture of five OTA-producing strains, whereas the most susceptible variety was Cabernet Sauvignon [46]. In the case of coffee, also only limited data are available regarding the resistance of different cultivars to OTA accumulation. However, intensive research has been carried out to identify and breed cultivars resistant to the attack of coffee berry borer [47], which presumably will lead to lower OTA levels in these coffee cultivars. Some *Coffea* species are naturally resistant to the coffee berry borer, with *Coffea abeokutae*, *Coffea excelsa,* and *Coffea kapakata* being the most resistant [48]. 

Other preharvest management approaches, including biocompetitive exclusion or genetic engineering, which has been successfully used to lower aflatoxin levels of corn, cotton, and peanuts, and fumonisin levels in corn, respectively, have not yet been applied in practice to lower ochratoxin levels in agricultural products [49]. 

### 2.2. Postharvest management

Although the prevention of mycotoxin contamination in the field is the main goal of the agricultural and food industries, the contamination of various commodities with *Aspergillus* or *Penicillium* isolates and their mycotoxins is unavoidable under certain environmental conditions. Postharvest strategies aim at lowering fungal contamination and consequently, the mycotoxin content of agricultural products during storage, handling, processing, and transport. Such strategies include the improvement of drying and storage conditions, the use of chemical and natural agents, and irradiation. 

During harvest, the following factors influence OTA contamination of cereals [50]: weather before and during harvest, time before drying, efficiency of drying machinery, physical state of grains, temperature at harvest, fungal competition, cleanliness of harvesters, and transport. Once in store, cereals must be regularly inspected and kept under safe storage conditions. Some important factors for keeping grain safe are: cleanliness of storage containers, absence of structural leaks, condensation, and temperature [50].

Regarding grape products, the code put forward by the Office International de la Vigne et du Vin (OIV) of sound viticultural practices for the minimization of OTA levels in vine-based products includes hygiene of the containers, measures to avoid fruit fly infestation, avoidance of overstacking, sorting, and drying conditions [51]. To decrease OTA content in wines, the removal of rotten grapes prior to crushing and pressing should be carried out. Since the OTA content of damaged berries was higher than that of undamaged ones, selecting grapes seems to be the best and natural way to limit OTA occurrence in wine [52]. 

In coffee, managing the risk of OTA contamination involves key factors, including good hygiene practices along the production chain, rapid drying, and avoiding the re-wetting of coffee by ensuring clean and dry storage and transportation (http://www.coffee-ota.org/4_1_prevention.asp). Coffee postharvest manufacturing is carried out using two processes. The dry method consists of a natural drying stage (in the sun) or an artificial drying stage, followed by mechanical dehulling. In the wet method, cherries are pulped, and the resulting beans are dried and dehulled. Pulping does not result in any significant change in the OTA content. However, there is a difference between fermentative and physical mucilage removal, the latter resulting in a substantial drop in OTA levels. After hulling, only traces are found in both cases. Afterwards, recontamination during the storage stage could lead to OTA production and accumulation [53].

Mycotoxin production is dependent on a number of factors, e.g., water activity of the stored product, temperature, gas composition, the presence of chemical preservatives, and microbial interactions. An integrated approach for controlling several of these factors could provide a much more effective control of deterioration without requiring extreme control of any one factor. Water availability or moisture content is one of the most important factors in the prevention of fungal growth and mycotoxin production [54]. It has been observed that grain stored at a moisture content equivalent to a water activity of 0.70 (<14.5% moisture by weight) or less will not be subject to spoilage and mycotoxin formation [13]. Temperature also influences fungal contamination during storage.

Some chemical preservatives, such as potassium sorbate or calcium propionate, are capable of preventing OTA contamination in cereal products, including bread. [55,56]. Several other antimicrobial food additives have been found to inhibit the growth or OTA production, or both, of fungi, including methyl para-hydroxybenzoic acid, propyl-paraben, and sodium propionate [57,58]. A new strategy under study is the use of antioxidants, such as vanillic acid or 4-hydroxybenzoic acid [59], and essential oils extracted from plants, such as *Thymus vulgaris*, *Aframomum danielli* [60,61], cinnamon, and clove leaf [62], which affect mold growth and OTA synthesis. Other essential oils extracted from plants (thyme, cinnamon, marigold, spearmint, basil, quyssum, caraway, and anise) [63], and those of oregano, mint, basil, sage, and coriander, have also been found to inhibit the growth of ochratoxigenic fungi, while oregano and mint oils also inhibited OTA production in an *A. westerdijkiae* isolate [64]. It was suggested that the inhibitory effects exerted by spices and herbs may rely, at least in part, on phenolic compounds, such as coumarins and flavonoids [65]. Indeed, flavonoids, including rutin, quercetin, and caffeic acid were found to inhibit both growth and OTA synthesis in *A. carbonarius* [66]. Additionally, alkaloids produced by *Piper longum*, and components of sesame oil and turmeric have also been found to suppress both fungal growth and ochratoxin production in a number of OTA-producing aspergilli [67]. 

Several reports have dealt with the antifungal properties of various lactic acid bacteria [68]. These bacteria are of special interest as biopreservation organisms because they have a long history of use in food and have been designated or generally regarded as safe. Lactic acid bacteria produce antimicrobial compounds, including organic acids, like lactic acid and acetic acid, hydrogen peroxide, cyclic dipeptides, phenyllactic acid, 3-hydroxylated fatty acids, bacteriocins, and low-molecular-weight proteinaceous compounds. Additionally, these bacteria compete with other species by acidifying the environment and rapidly depleting nutrients. They are also promising tools for controlling ochratoxigenic fungi in various food products [68]. 

Another possibility of inhibiting the growth of mycotoxigenic fungi is the application of modified atmospheres or gases, such as CO_2_ [69], N_2_, CO, and SO_2_ for the protection of cereal grain from fungal spoilage and mycotoxin contamination during the postharvest period. Modified atmosphere storage has been examined for the storage of moist grain, especially for animal feed. Studies with *P. verrucosum* and *A. ochraceus* with up to 50% CO_2_ suggest that spore germination is not markedly affected, although germ tube extension, and hence colonization, is significantly inhibited by 50–75% CO_2_, especially at water activities above 0.90 [69]. Paster *et al.* [70] reported that OTA production by *A. ochraceus* was completely inhibited by >30% CO_2_ on agar-based media after 14 days, suggesting that there are differences between mycotoxigenic species. This suggests that for efficient storage of moist cereals CO_2_ concentrations of >50% need to be achieved rapidly to prevent OTA contamination in storage or during transport [62]. Postharvest control of grape rot caused by black aspergilli (among other fungi) has also been successfully carried out using acetaldehyde vapors [71]. 

Irradiation of fresh fruits, including grapes and figs, can significantly decrease fungal counts [72]. γ-irradiation has also been used successfully to decompose OTA in liquid media [73].

Recently, fungicide treatment of stored products have also been claimed as a promising tool for the prevention of OTA accumulation in agricultural products [74]. A particularly effective treatment for cereals, nuts, fruits, and spices was suggested to be a combination of fungicides, e.g., prothioconazole with trifioxystrobin, tebuconazole with trifloxystrobin, or tebuconazole with prothioconazole.

### 2.3. HACCP approaches

Knowledge of the key critical control points during the harvesting, drying, and storage stages of the cereal production chain is essential for the development of effective pre- and postharvest prevention strategies. Ecological studies on the effect of environmental factors, which are marginal for growth and mycotoxin production, have been identified for *P. verrucosum* and *A. ochraceus* in relation to cereal production and for *A. carbonarius* in relation to grapes and wine production [75]. Magan [75] and Magan and Aldred [62] gave detailed accounts of pre- and postharvest control strategies for mycotoxin contamination of food in the context of an HACCP (**H**azard **A**nalysis and **C**ritical **C**ontrol **P**oints) framework. Critical control points were identified at different stages of the coffee, cereal, and wine production chains, and these HACCP approaches have been used successfully to control OTA levels in these agricultural products [25,62,75,76,77].

## 3. Decontamination/Detoxification Approaches

The ideal solution for reducing the health risk of mycotoxins is to prevent contamination of foods with them. Unfortunately, contamination cannot be completely avoided. Therefore, there is an increased focus on effective methods of detoxification for mycotoxins present in foods, and on the inhibition of mycotoxin absorption in the gastrointestinal tract. Decontamination or detoxification procedures are useful in order to recondition mycotoxin contaminated commodities. While certain treatments have been found to reduce levels of specific mycotoxins, no single method has been developed that is equally effective against the wide variety of mycotoxins, which may co-occur in different commodities. Mycotoxin decontamination processes should meet the following criteria [12]:

- They must destroy, inactivate, or remove mycotoxins;
- They must not produce or leave toxic, carcinogenic, or mutagenic residues in the final products or in food products obtained from animals fed by decontaminated feed;
- They should not adversely affect the desirable physical and sensory properties of the product;
- They must be capable of destroying fungal spores and the mycelium in order to avoiding mycotoxin formation under favorable conditions;
- They have to be technically and economically feasible.

- Several strategies are available for the detoxification or decontamination of commodities containing ochratoxins. These can be classified as physical, physicochemical, chemical, and (micro)biological approaches.

### 3.1. Physical methods

In the European Union, dilution with non-contaminated foodstuffs is forbidden. The physical methods used for mycotoxin detoxification include cleaning, mechanical sorting, and separation (e.g., filtering), heat treatment, ultrasonic treatment, and irradiation. During the cleaning process of contaminated grain, dust, husks, hair, and shallow particles are separated from the grain. During mechanical sorting and separation, the clean product is separated from mycotoxin-contaminated grains, while washing procedures using water or sodium carbonate solution can also result in some reduction of mycotoxins in grains. OTA is a moderately stable molecule. It can survive most food processing, such as roasting, brewing, and baking to some extent [78,79]. Ensiling was found to reduce the OTA content of barley [80]. The scouring of wheat can lead to a reduction of more than 50% in OTA concentration, while milling hard wheat to produce white flour resulted in an approximately 65% reduction, and a further 10% decrease occurred during baking [81].

OTA is generally stable at temperatures used during ordinary cooking. Boudra *et al.* [82] showed that OTA is heat stable, and it took for more than 10 hours (700 min) and 200 min to decompose 50% of OTA in dry wheat at 100 °C and 150 °C, respectively. However, due to the high temperatures used for coffee roasting, a higher percentage of destruction was observed, although contradictory results from different studies have been reported. Initial studies on the influence of the roasting process on OTA levels indicated a reduction of 77–87% [83], 80–90% [84], and 90–100% [85], although opposing values of 0–12% [86] and 2–28% [87] have also been published. These differences can be due to different spiking methods, selectivity and sensitivity values, initial contamination level, and roasting and drying conditions, or inhomogeneous toxin distribution. More recently, Blanc *et al.* [88] have found a loss of 84%, van der Stegen *et al.* [89] of more than 69%, Romani *et al.* [90] of more than 90%, and Pérez de Obanos *et al.* [91] of 13–93%. Urbano *et al.* [92] found that roasting at the temperature of 200 °C for 10 minutes reduced OTA content only by 22%. However, by increasing the temperature to 220 °C for 15 minutes, the OTA content was reduced by up to 94%. Ferraz *et al.* [93] observed a 8–98% OTA reduction in artificially contaminated coffee beans, depending on the temperature and time period of roasting. The spouted bed roasting used by these authors proved to be a very efficient procedure for OTA reduction in coffee, and its reduction depended directly on the degree of roasting. They also suggested that the low reduction in OTA content observed by some previous authors could be due to the use of a static oven, in which heat exchange is very low. In such a situation, a considerable part of the roasting time might have been used just for drying, with the beans remaining at temperatures of around 80–90 °C. Three different explanations were suggested for this reduction: physical OTA removal with the husk, isomerization at the C-3 position into another diastereomer, and thermal degradation with the possible involvement of moisture [89].

**Table 1:** OTA reduction during coffee roasting.

Origin of OTA	Roasting conditions	Reduction in OTA content (%)	References
Inoculation	200 °C, 10–20 min	0–12	[86]
Inoculation	180 °C, 10 min	31.1	[94]
Natural	200 ± 5 °C, 20 min	77–87	[83]
Natural	200 ± 5 °C, 20 min	80–90	[84]
Natural	200 °C, 3 min	65–100	[95]
Inoculation	180–240 °C, 5–12 min	8–98	[93]
Inoculation	5–6 min, dark roasting	48–87	[85]
Natural	5–6 min, dark roasting	90–100	[85]
Natural	250 °C, 150 sec	14–62	[87]
Inoculation	250 °C, 150 sec	2–28	[87]
Natural	223 °C, 4 min	84	[88]
Natural	Light to dark	69–96	[89]
Natural	175–204 °C, 7–9 min	>90	[90]
Inoculation	200–220 °C, 10–15 min	22.5–93.9	[92]
Natural	Industrial roasting	66.5	[91]

Regarding other coffee processing techniques, Wilkens and Jörissen [96] showed that cyclone cleaning of green coffee beans had little effect on the beans’ OTA concentration: although the OTA concentration in the discarded fraction was high, the dust comprised <1% of the weight of the cleaned coffee. Sorting with color sorters resulted in some reduction, and steaming caused a mean 25% reduction. During coffee brew manufacturing, the coffee grinding entails OTA losses of 20% [88]. Coffee steaming might also promote OTA removal (reduction of about 25%) [97]. Regarding brew preparation, contradictory studies exist: some authors found that all the toxin found in the coffee bean was still present in the brew [86,89], while others noted that 90–100% of the OTA was absent after this stage [85]. More recently, Pérez de Obanos *et al.* [91] have pointed out that, depending on the brew preparation method used, the OTA losses vary in the range 15–50%. Heilmann *et al.* [97] studied OTA reduction in raw coffee beans roasted industrially, and showed that levels of OTA were significantly reduced, especially in coffee decaffeinated by solvent extraction. Similar results were reported by Micco *et al.* [85].

A freezing (−20 °C) defrost (26 °C) process and UV and γ irradiation treatments were found to be able to destroy the conidia of mycotoxin producing fungi, but only γ-irradiation was found to destroy OTA itself [72,98,99,100]. 

Wine filtration through a 0.45 μm membrane showed an 80% decrease of OTA [101]. More recently, an environmentally friendly corrective measure has been developed to reduce OTA levels by repassage of contaminated musts or wines over grape pomaces having little to no OTA contamination [102]. The use of grape pomaces from red wines of the same grape variety did not affect wine quality parameters, including color intensity and health-promoting phenolic content. 

Extensive detoxification of mycotoxins, including ochratoxins, could be achieved by using the intermittent ultrasonic treatment of cereal grains in an aqueous medium supplemented with alcohol and alkali in a temperature range of 12–50 °C [103,104]. The decontaminated cereal products or treated products remained largely unchanged with respect to their appearance, flavor, and nutritious value.

### 3.2. Physicochemical methods

Another approach for removing mycotoxins from contaminated agricultural products involves the use of adsorbent materials with the capacity to tightly bind and immobilize mycotoxins. Adsorbing agents can be classified into different groups based on their origin: minerals (e.g., aluminosilicates), activated coals, biological adsorbents (e.g., yeast and bacterial cell walls or vegetal fibers), and synthetic adsorbents, including modified natural clays (e.g., grafting of quaternary ammonium groups) and synthetic resins (e.g., polyvinylpyrrolidone, cholestyramine). Such materials have been tested mainly to lower OTA contamination of wine and must. Several fining agents have been tested for their ability to remove OTA from contaminated must or wines [26,105]. Activated carbon and potassium caseinate have been reported to remove the highest amounts of OTA, although carbon also removes anthocyanins and other colored polyphenols from wine. Potassium caseinate was able to remove up to 82% of OTA when used at 150 g hl^−1^, while activated carbon showed the highest specific adsorption capacity, owing to its high surface area per mass ratio, and the low adsorption of total polyphenols. Dumeau and Trione [106] achieved reductions in the OTA content of red wines of up to 90%, although maximum reductions led to negative effects on wine quality, such as reduction of the color intensity and of the polyphenol index of wines. Products like gelatine preparations, silica gel powder, even cellulose, gave good results. Enological decolorizing carbon was also able to remove up to 72% of OTA in red wines during a recent study carried out by Gambuti *et al.* [101], and the carbon did not affect either polyphenol content or the color of wine, although at this time, a decrease of several sensory odorants was observed. The effectiveness of treatment with oak wood fragments depended upon the quantity of wood chips and powder used [107]. According to the results of Olivares-Marin *et al.* [108], activated carbon produced from cherry stones could be used to remove up to 50% of OTA from wines, and the changes produced in the total polyphenol index and color intensity were small. Modified silica gels, dodecylammonium bentonite, KSF-montmorillonite, and chitosan beads have also been found to be useful for OTA removal from wine [109,110]. Other adsorbents, including bentonite, cellulose acetate esthers, polyvinylpyrrolidone, cholestyramine, and polygel were inefficient for OTA removal [26,101,111]. 

Bacteria, for example, *Lactobacillus plantarum* and *Oenococcus oeni*, have been found to reduce thre OTA content of wine [52,112]. However, later Mateo *et al.* [113] observed that part of the OTA was not sorbed by *O. oeni* and remained in the liquid medium as on ethanol-containing media. Thus, the bacteria cannot efficiently eliminate OTA in acidic ethanol-containing beverages, such as wine. The addition of these adsorbents to red wine by up to 20 g hl^−1^ did not modify substantially the color, but high amounts of adsorbent (≥50 g hl^−1^) affected the color and the organoleptic properties of wines [52]. Bejaoui *et al.* [114] successfully used inactivated *Saccharomyces* strains to lower the OTA content of grape juices. Their results showed that treatments of yeasts by heating and acids significantly enhanced OTA removal from liquid media. Polysaccharides and peptidoglycans are both expected to be affected by heat and acid treatments, and the released products could offer more adsorption sites than viable cells and may increase surfaces for OTA binding. Yeast cell wall preparations and other yeasts have been found to be effective for OTA adsorption by other authors too [115,116]. There are two main classes of proteins covalently coupled to cell wall polysaccharides in *S. cerevisiae*, among these, GPI-dependent cell wall proteins, which are generally indirectly linked to β-1,3-glucan through a connecting β-1,6-glucan moiety, have been suggested to be responsible for OTA adsorption on the cells wall [117,118,119]. Raju *et al.* [120,121] have shown that OTA also binds to the glucomannan component of the cell wall. Yeast industrial byproducts have also been used successfully to bind OTA from wine [122,123]. 

In conclusion, many adsorbent materials have been tested for OTA detoxification; however, their activity was not as high as expected, with the exception of activated charcoal [79,124,125]. Recently, a new insoluble vegetal fiber has been developed, which is found to be able to adsorb OTA present in liquid food products [126]. Other promising adsorbent materials are modified zeolites, as they have shown good results in foodstuff decontamination [127,128,129,130]. Organophilic bentonites showed the ability to bind OTA up to 100% independently of the pH of the test medium buffer [131].

### 3.3. Chemical approaches

A wide variety of chemicals have been found to be effective in destroying mycotoxins. The chemicals used include various acids, bases, oxidizing agents, chlorinating or reducing agents, salts, and miscellaneous reagents, such as formaldehyde. Ammoniation is the method that has received the most attention for detoxification of aflatoxin- or ochratoxin-contaminated feeds and has been used successfully in several countries [132]. Ammoniation almost completely decomposes OTA in corn, wheat, and barley [79]. Although the ammoniation process does not lead to the formation and accumulation of toxic breakdown products of mycotoxins in agricultural products, the observed changes in sensory and nutritional qualities (e.g., the brown color of the treated cereals and a decrease in lysine and sulfur-containing amino acids), the relatively long period of aeration, and the cost restrict its use in cereals destined to be used in animal feed formulations [79,133]. Besides, in feeding experiments, some toxicity and lower nutritional values were observed when ammoniated OTA-contaminated barley was used [134]. Consequently, ammoniation was not recommended for the detoxification of OTA-contaminateed feeds [79]. However, ammoniation is an approved procedure for the detoxification of aflatoxin-contaminated agricultural commodities and feeds in some US States (e.g., in Arizona, California, Texas, Georgia, and Alabama). Additionally, in France and Senegal, ammoniation is used for mycotoxin detoxification in contaminated peanut, cotton, and maize meals [135,136,137]. The import of ammoniated peanut meal is allowed by several European Union member countries. However, the US Food and Drug Administration (FDA) does not permit interstate shipment of ammoniated cottonseed or maize.

Alkaline hydrogen peroxide, sodium hydroxide, and monomethylamine or ammonium with calcium hydroxide treatments have also been found to be effective methods for OTA decontamination in this matrix [79]. Nevertheless, their *in vivo* application is not possible due to the undesirable changes caused in the nutritional and sensory quality of the substrate [79]. 

Regarding other agricultural products, treatments with ethyl acetate, dichlorometane, and methylene chloride supplemented with 2% formic acid were found to be able to reduce OTA levels by up to 80% in coffee beans [97,138,139]. In cocoa, more than 98% of the OTA could be eliminated by alkaline treatment [140]. Another method is ozonization; the development of sophisticated electrochemical techniques has allowed the application of ozone (O_3_) for OTA removal up to undetectable levels in foodstuffs such as grains, nuts, or vegetables [141,142]. OTA was degraded in 15 sec using 10 wt.% O_3_ in an aqueous solution, with no by-products detectable by HPLC [142].

Formic, propionic, and sorbic acids and sodium hypochlorite at concentrations ranging from 0.25% to 1% have also been found to degrade OTA after an exposure of 3–24 hours [79,143]. However, alkaline hydrogen peroxide treatment with 0.05–0.1% H_2_O_2_ did not detoxify OTA [144]. We should mention that chemical treatment is not allowed within the European Union for commodities destined for human consumption. An alternative strategy could be the utilization of microorganisms capable of detoxifying mycotoxins in contaminated foods and feeds (see below).

### 3.4. Microbiological methods

Microbes or their enzymes can be applied for mycotoxin detoxification; such biological approaches are widely studied [12,129,135,137,145,146,147]. Several reports describe the OTA degrading activities of the microbial flora of the mammalian gastrointestinal tract, including the rumen microbes of cows and sheep [148,149], and the bacteria that mainly live in the caecum and large intestine of rats [150,151]. The duodenum, ileum, and pancreas have also been shown to be able to carry out this reaction in rats, whereas the activity in the liver and kidney was low [152], and was not observed in rat hepatocytes [153], nor in rabbit and rat liver [154]. Up to 12 mg kg^−1^ of OTA in feed was estimated to be converted to ochratoxin α (OTα) in cows [149]. Thus, this species is assumed to be relatively resistant to the effects of OTA in feed. Sheep also have a good capacity to detoxify OTA before it reaches the blood [155]. Studies in mice suggested that OTA circulates from the liver into the bile and into the intestine, where it is also hydrolysed to OTα [156]. The species responsible for OTA detoxification have not yet been identified, although protozoa were suggested to take part in the biotransformation process in ruminants [155,157]. The enzymes responsible for hydrolysis to OTα in cows and rodents are carboxypeptidase A and chymotrypsin [158]. The human intestinal microflora can also partially degrade OTA [159]. 

**Figure 1:**
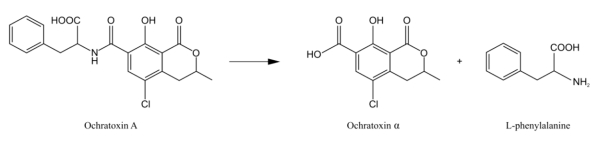
Degradation of ochratoxin A by carboxypeptidase A.

Additionally, numerous other bacteria, protozoa, and fungi have been found to be able to degrade OTA since the 80s (Table 2). Some enzymes, such as carboxypeptidase A [158,160,161], lipases from *Aspergillus niger* [162], and some commercial proteases [163] have also been identified as being able to carry out this reaction (Figure 1). 

*Acinetobacter calcoaceticus* has also been found to degrade OTA to OTα [164]. These authors hypothesize that an extracellular esterase enzyme is responsible for OTA degradation. The toxicity of OTA decreased since OTα is almost nontoxic [165,166], although it has been found to exhibit genotoxic effects [167]. Actually, most OTA degrading microbes have been found to be able to remove the phenylalanine moiety from OTA, which leads to the accumulation of OTα. *Rhizopus* isolates are also able to partially degrade OTA within ten days. However, only a *R. stolonifer* isolate could detoxify OTA in spiked moistened wheat [168]. More recently, Angioni *et al.* [169] observed that *Saccharomyces cerevisiae* and *Kloeckera apiculata* isolates were able to degrade OTA during alcoholic fermentation. The absence of OTA residues in the biomass excluded an adsorbing effect from the yeast cell walls of the strains studied, and the absence of ochratoxin α and phenylalanine suggested other degradation pathways of OTA than what was observed in most other microorganisms. Silva *et al.* [170] also observed the OTA degrading activities of a *Lactobacillus plantarum* isolate. However, degradation products of OTA were not observed, indicating that possibly OTA adsorption, instead of degradation, took place in these cases. OTA was also efficiently detoxified by some *Bacillus* isolates, especially by *B. licheniformis* CM21 [171]. Similarities between OTA degradation kinetics by *Aspergillus niger* and *Bacillus* isolates and the detection of the degradation product, OTα in the ferment broth of *B. licheniformis* suggest that carboxypeptidase A activity may be responsible for OTA decomposition by these isolates [171]. 

Several aerobic or anaerobic bacteria and yeasts have been identified, which were found to cleave the phenylalanine group of the ochratoxins [147,172,173] (Table 2). Among these microbes, the detoxifying bacteria belonging to *Sphingomonas* sp., *Stenotrophomonas nitritreducens*, *Stenotrophomonas*, *Ralstonia eutropha*, and *Eubacterium* sp., and the detoxifying yeasts belonging to *Trichosporon sp.*, *Cryptococcus* sp., *Rhodotorula yarrowii*, *Trichosporon mucoides*, and *Trichosporon dulcitum* have proved to be particularly efficient, since they not only ensured complete degradation of OTA, but could additionally be used safely in food products and animal feeds, which is not necessarily the case with a plurality of other mycotoxin-cleaving and/or degrading bacteria and yeasts.

**Figure 2:**
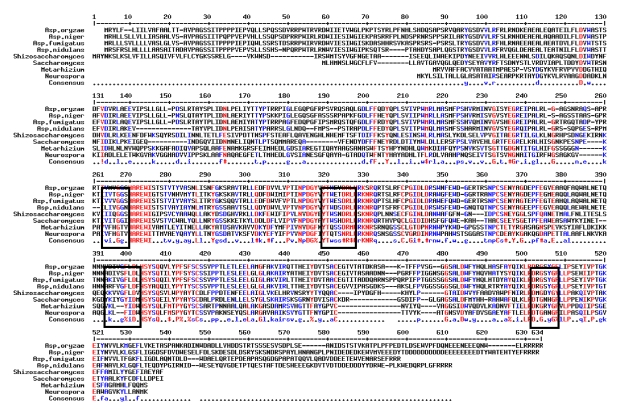
Comparison of the partial amino acid sequences of the carboxypeptidase A (*cpa*) gene of *Aspergillus niger* to those of metalloproteases from other fungi. Black boxes indicate highly conserved domains coding for Zn ion binding sites.

*Streptococcus salivarius*, *Bifidobacterium bifidum*, *Lactobacillus delbrueckii*, and yogurt bacteria have completely reduced OTA levels in milk samples [174]. Varga *et al.* [175] examined more than 70 *Aspergillus* isolates for their ability to degrade OTA to OTα, which has only limited toxicity. Only *A. fumigatus* and some black *Aspergillus* isolates were found to be able to carry out this reaction. The kinetics of the degradation of OTA of an atoxigenic *A. niger* strain was further studied. OTA degradation was faster in solid media than in liquid cultures. *A. niger* could also degrade ochratoxin α to an unknown compound within some days [175]. This is a promising result because it might allow for the biological elimination of this mycotoxin and may provide a source of enzymes, which could be used for detoxification of OTA in contaminated agricultural products. Further studies have been carried out to identify the enzyme responsible for OTA degradation in *A. niger* CBS 120.49 [176]. It was predicted that the enzyme involved in the reaction is possibly a carboxypeptidase, as carboxypeptidase A can convert OTA to ochratoxin α. The entire *cpa* gene in *A. niger* was identified and the promoter and terminal regions of this gene were also determined. The whole *cpa* gene has been cloned and analyzed: a 3287 base pair (bp) long sequence was determined. This sequence contains a 1939 bp long open reading frame and a 72 bp long intron. This open reading frame encodes a 621 amino acid long protein (Figure 2). Besides the coding region, the 648 bp long promoter region and the 700 bp long terminal region were determined as well. In further experiments, the gene was transformed to a *Pichia pastoris* isolate; the isolated *cpa* gene was inserted into the pPCIZα vector, which was used in the transformation of the *Pichia pastoris* KM71H isolate. The results showed that this protein was not secreted into the ferment broth or was present only in low quantities. Experiments have also been initiated to transform the gene into an atoxigenic *A. niger* (JHC 607) and *A. nidulans* (SZMC 0552) isolate, without success. The results showed that these transformants were unable to degrade OTA in liquid medium. We suppose that the *cpa* gene was not integrated into the genome of *A. niger* and *A. nidulans*, or was not expressed in these isolates. Further studies are in progress to clarify the role of this gene in OTA degradation.

**Table 2:** Microbes and enzymes able to degrade ochratoxin A.

**Microbes or enzymes**	**Reference**
**Bacteria**	
Rumen microbes	[149, 175]
*Butyrivibrio fibrisolvens*	[187]
*Lactobacillus*, *Streptococcus*, *Bifidobacterium* sp.	[174]
*Bacillus subtilis*, *B. licheniformis*	[171,188]
*Acinetobacter calcoaceticus*	[164]
*Phenylobacterium immobile*	[189]
*Nocardia corynebacterioides*, *Rhodococcus erythropolis*, *Mycobacterium sp.*	[190]
*Lactobacillus* sp.	[191,192]
*Eubacterium callenderi*, *E. ramulus*, *Streptococcus pleomorphus*, *Lactobacillus vitullinus*, *Sphingomonas paucimobilis*, *S. saccharolytica*, *Stenotrophomonas nitritreducens*, *Ralstonia eutropha*, *R. basilensis*, *Ochrobactrum* sp., *Agrobacterium* sp.	[147,172]
*Pseudomonas cepacia*, *P. putida*, *Rhodococcus erythropolis*, *Agrobacterium tumefaciens*, *Comomonas acidovorans*	[173]
**Protozoa**	[155,157]
**Fungi**	
*Aspergillus niger*, *A. fumigatus*	[175]
*Aspergillus niger*, *A. versicolor*, *A. wentii*, *A. ochraceus*	[177]
*Aspergillus niger*, *A. japonicus*	[178]
*Pleurotus ostreatus*	[193]
*Saccharomyces cerevisiae*	[191,194]
*Saccharomyces cerevisiae*, *S. bayanus*	[114]
*Rhizopus stolonifer*, *R. microsporus*, *R. homothallicus*, *R. oryzae*	[168]
*Trichosporon mycotoxinivorans*	[195]
*Phaffia rhodozyma*, *Xanthophyllomyces dendrorhous*	[180]
*Saccharomyces cerevisiae*, *Kloeckera apiculata*	[169]
*Aureobasidium pullulans*	[39]
*Cryptococcus flavus*, *C. laurentii*, *C. curvatus*, *C. humicolus*, *Trichosporon ovoides*, *T. dulcitum*, *T. guehoae*, *T. mucoides*, *T. coremiiforme*, *T. cutaneum*, *T. laibachii*, *T. monilifotrme*, *Rhodotorula mucilaginosa*, *R. fujisanensis*	[173,196]
**Enzymes**	
Carboxypeptidase A	[158,161]
Commercial proteases (Pancreatin from porcine pancreas, Protease A and Prolyve PAC from *A. niger*)	[163]
Commercial hydrolases (Amano A, crude lipase preparation from *A. niger*)	[162]
*A. niger* hydrolytic metalloenzyme	[197]

Abrunhosa *et al.* [177] identified 51 fungal isolates from grapes with the ability to degrade more than 80% of the OTA added to a culture medium. The most effective isolates belonged to the *A. clavatus*, *A. ochraceus*, *A. versicolor*, and *A. wentii* species. Other genera found to degrade OTA include *Alternaria*, *Botrytis*, *Cladosporium*, and *Penicillium* [177]. In the cases of *A. ochraceus* and *A. wentii*, OTα was not detected in the medium as a breakdown product of OTA [177]. Similarly, some strains of *A. carbonarius*, *A. niger* aggregate, and *A. japonicus* isolated from French grapes degraded more than 80% of the OTA to OTα in liquid medium [178]. Some yeasts belonging to the genera *Rhodotorula*, *Cryptococcus*, and *Pichia* have also been found to be able to degrade OTA [179]. 

We recently examined the OTA degrading and adsorbing activities of astaxanthin-producing yeast isolates (*Phaffia rhodozyma* and *Xanthophyllomyces dendrorhous*) [180]. The data indicate that besides producing astaxanthin, *Ph. rhodozyma* is also able to both detoxify and adsorb OTA at temperatures well above the temperature optimum for the growth of *Phaffia* cells. One *Ph. rhodozyma* and two *X. dendrorhous* isolates have been tested for OTA degradation. All of them were able to degrade OTA in a liquid medium after less than 10 days. In further studies, we concentrated on the OTA degradation or adsorbing activities of the isolate, CBS 5905 of *Ph. rhodozyma*. This *Phaffia* isolate could degrade more than 90% of the OTA in about 7 days at 20 °C. Interestingly, a significant amount of OTA was found to be bound by the cells after two days, indicating that the OTA is also adsorbed by the cells. The ferment broth of either induced or uninduced cells was unable to degrade OTA. These observations indicate that the enzyme responsible for OTA degradation is not excreted into the ferment broth; thus, the enzyme responsible for OTA degradation is cell-bound. Our data indicate that *Ph. rhodozyma* is able to convert OTA to OTα, and that this conversion is possibly mediated by an enzyme related to carboxypeptidases. Chelating agents like EDTA and 1,10-phenanthroline inhibit the OTA degradation caused by *Ph. Rhodozyma*, indicating that the OTA degrading enzyme, like carboxypeptidase A, is a metalloprotease. The temperature optimum of this enzyme was found to be above 30 °C, which is much higher than the temperature optimum for the growth of *Ph. rhodozyma* cells, which is around 20 °C. Both viable and heat-treated (dead) *Ph. rhodozyma* cells were also able to adsorb significant amounts of OTA. Further studies are in progress to identify the enzyme responsible for OTA degradation in *Ph. rhodozyma* [176].

Cell cultures of plants like maize, tomato, and wheat have been found to be able to transform OTA into a number of compounds. The transformation reactions included hydrolysis of ester and peptide bonds, methylation and hydroxylation, some of which led to a loss in toxicity [181,182,183,184]. OTA was found to be toxic to several invertebrates, such as the corn ear worm (*Helicoverpa zea*) and *Carpophilus hemipterus*, while others, e.g., the Mediterranean flour moth (*Anagasta kuehniella*) were not affected [185]. Since these insensitive animals possibly produce some factors which are able to inactivate OTA, they are regarded as promising sources of OTA detoxifying enzymes [184].

Based on an EFSA survey, *Trichosporon mycotoxinivorans* was found to be the only microorganism that shows the potential to degrade OTA and meets the prerequisites for use as an animal feed additive [186].

## 4. Prevention of Toxic Effects of OTA

It is also possible to prevent the toxic effects once OTA is ingested. Sodium bicarbonate is efficient at preventing absorption through the stomach [165]. The toxicity of OTA can be reduced by the simultaneous administration of phenylalanine or by pretreatment with phenobarbital in mice [156]. The addition of phenylalanine also prevented the immunosuppressive effects of OTA [198]. However, addition of phenylalanine had no effect on the neurotoxicity of OTA in embryonic chick brain cell cultures [199], and did not decrease either the toxicity or carcinogenicity of OTA in chicken [200,201]. Besides, phenobarbital increased OTA-induced hepatocarcinogenicity in mice [156]. In addition, both phenylalanine and phenobarbital have been shown to increase OTA genotoxicity in cell culture [202]. The phenylalanine analog, aspartame (L-aspartyl-L-phenylanine methyl ester), and piroxicam, a non-steroidal anti-inflammatory drug, effectively prevented OTA-induced toxic effects in rats [203]. Aspartame is widely used as a sweetener, and possibly inhibits OTA-induced toxicity by preventing OTA from binding to plasma proteins. Studies on monkey kidney cells showed that aspartame prevents or partially protects against some typical cytotoxic effects of ochratoxin, such as the inhibition of protein synthesis, lipid peroxidation, and the leakage of certain enzymes, such as lactate dehydrogenase, gamma-glutamyl transferase, and alkaline phosphatase [204]. *In vitro*, aspartame prevented ochratoxin from binding to plasma proteins.When given to rats, aspartame prevented ochratoxin induced genotoxicity and nephrotoxicity and washed out the toxin from the body efficiently. The protective action is thought to be due to its structural similarity to phenylalanine and ochratoxin. Some authors claim that aspartame is the best candidate for preventing ochratoxin-induced subchronic effects [133].

Adsorbents have also been tested as potential binding agents for lowering OTA levels in body fluids. Phillips *et al.* [205] showed that hydrated sodium calcium aluminosilicates (HSCAS) have a high affinity for aflatoxin B_1_ after screening 38 different adsorbents that were representative of the major chemical class of aluminas, silicas, and aluminosilicates, although the efficacy of HSCAS was quite limited against OTA [125,206]. However, a feed additive resulting from the modification and activation of diatomaceous earth was used successfully as a feed additive in laying hens [207].

Activated carbons exhibited high *in vitro* affinity to OTA [208], although addition of charcoal to the diet of chicken did not reduce OTA toxicity significantly [209]. However, a carbon/aluminosilicate-based feed additive has been found to bind OTA effectively in a model of the porcine gastrointestinal tract [210]. Micronized wheat fibers have also been used successfully as feed additives in piglets to counteract the effects of OTA [211]. Esterified glucomannan was found to have a protective effect against OTA and T-2 toxin [120] and diminished the growth depression caused by a naturally contaminated diet in broilers [212]. However, later studies found that esterified glucomannan was ineffective in alleviating the toxic effects of OTA in minks [213]. Dried whole yeast cell mass, yeast cell wall extracts, and wall components of *Lactobacillus rhamnosus* have been observed to be able to bind mycotoxins *in vitro* [214]. Cholestyramine, a resin used for pharmaceutical purposes in decreasing total and LDL cholesterol, is able to adsorb zearalenone, aflatoxins, OTA, and fumonisins in *in vivo* experiments [215]. However, cholestyramine is too expensive to be used in farms efficiently. In conclusion, although several adsorbents have been developed for lowering the toxic effects of mycotoxins as feed additives, most of them were found to be ineffective against OTA-induced toxicity in *in vivo* tests [206]. 

Since OTA is known to cause cell membrane damage through increased lipid peroxidation, the protective properties of antioxidant substances have also been investigated. Aspirin and indomethacin were found to protect mice from the genotoxic effects of OTA [216], while antioxidants like retinol, ascorbic acid, and vitamin E prevented OTA-induced DNA adduct formation in laboratory animals [217,218]. *In vitro* and *in vivo* studies showed that melatonin [219], *N*-acetyl-cystein [220], rosmarinic acid [221], α-tocopherol, retinol [222], cyanidin-3-*O*-β-glucopyranoside [223], vitamin A [224], vitamin E [225], L-methionine [226], catechins [227], combined antioxidants [228], various alkaloids [229], and *Emblica officinalis* aqueous extracts [230] counteract some of the toxic effects of OTA. 2-mercaptoethane sulfonate has been found to protect rats from nephrotoxicity by increasing the number of available free thiol groups in the kidney [231]. Other substances that could reduce the damages caused by this mycotoxin in chicks and laying hens are roxazyme-G, sesame seed, water extract of artichoke, and L-phenylalanine [200,201]. Sesame seeds give strong protection against OTA-induced suppression of the humoral immune response, for which artichoke also has some beneficial effect, whereas phenylalanine has hardly any effect. Additionally, enzymes like superoxide dismutase and catalase were also found to prevent OTA-induced renal lesions in rats [232].

Many researchers have reported the protective effects of probiotic bacteria against OTA-induced toxicity. The probiotic bacteria found to be effective against OTA include *L. rhamnosus* [214], *L. acidophilus*, *Bifidobacterium longum* [192], *L. brevis*, *L. plantarum*, *L. mesenteroides* and *Oenococcus oeni* [112], *L. paracasei*, *L. Brevis*, and *L. plantarum* [233]. 

Dietary *Trichosporon mycotoxinivorans*, or a combination of *Eubacterium* BBSH 797 and *Trichosporon mycotoxinivorans* completely blocked OTA-induced immunosuppression in broiler chicks [234,235]. A feed additive named Mycofix® Plus contains *T. mycotoxinivorans*, mycotoxin adsorbents upgraded by the addition of epoxidase and lactonase activities, and is successfully used in broilers to counteract the toxic effects of OTA [236]. 

Animal feeding experiments with whole yeast cell, cell wall, or cell wall components have also shown positive results and reduced the mycotoxin toxicities of contaminated feeds in the investigated animals, although these results were not demonstrated for ochratoxins [237]. This indicates the stability of the yeast-mycotoxin complex through the gastrointestinal tract and shows the possibility of using *S. cerevisiae* as a detoxifying agent in the contaminated foods. 

## 5. Conclusions

Ochratoxins are among the most economically important of mycotoxins. Ochratoxin-producing fungi may contaminate agricultural products in the field (preharvest spoilage), during storage (postharvest spoilage), or during processing. Ochratoxin contamination of foods and feeds poses a serious health hazard to animals and humans. Due to OTA toxicity, and in order to assure human and animal health, this toxin is not allowed to be present above maximum permitted levels in agricultural products intended to be used as foods or animal feed. Substantial efforts have been exerted to study the critical points of OTA presence in the production chain of several affected commodities, including those of cereals, coffee, and wine, and detoxification methods have also been investigated. Pre- and postharvest OTA prevention strategies have been accepted as the most effective approach to managing contamination with this mycotoxin. Although it is not possible to entirely prevent the formation of OTA in all places, OTA accumulation can be minimized. There is also an increased focus on effective methods for detoxifying contaminated commodities. Decontamination or detoxification procedures are useful in order to recondition mycotoxin contaminated agricultural products for use as animal feeds. While certain treatments have been found to reduce levels of specific mycotoxins, no single method has been developed that is equally effective against the wide variety of mycotoxins, which may co-occur in different commodities. Regarding OTA, the most promising approaches include the use of microbes or their enzymes for decontamination purposes. Further extensive research is needed to identify more effective organisms that can be used safely for OTA decontamination.
